# Thymoquinone Suppresses IRF-3-Mediated Expression of Type I Interferons via Suppression of TBK1

**DOI:** 10.3390/ijms19051355

**Published:** 2018-05-03

**Authors:** Nur Aziz, Young-Jin Son, Jae Youl Cho

**Affiliations:** 1 Department of Integrative Biotechnology and Biomedical Institute for Convergence (BICS), Sungkyunkwan University, Suwon 16419, Korea; nuraziz@skku.edu; 2 Department of Pharmacy, Sunchon National University, Suncheon 57922, Korea

**Keywords:** thymoquinone, type I interferons, IRF-3, TBK1, inflammation

## Abstract

Interferon regulatory factor (IRF)-3 is known to have a critical role in viral and bacterial innate immune responses by regulating the production of type I interferon (IFN). Thymoquinone (TQ) is a compound derived from black cumin (*Nigella sativa* L.) and is known to regulate immune responses by affecting transcription factors associated with inflammation, including nuclear factor-κB (NF-κB) and activator protein-1 (AP-1). However, the role of TQ in the IRF-3 signaling pathway has not been elucidated. In this study, we explored the molecular mechanism of TQ-dependent regulation of enzymes in IRF-3 signaling pathways using the lipopolysaccharide (LPS)-stimulated murine macrophage-like RAW264.7 cell line. TQ decreased mRNA expression of the interferon genes *IFN-α* and *IFN-β* in these cells. This inhibition was due to its suppression of the transcriptional activation of IRF-3, as shown by inhibition of IRF-3 PRD (III-I) luciferase activity as well as the phosphorylation pattern of IRF-3 in the immunoblotting experiment. Moreover, TQ targeted the autophosphorylation of TANK-binding kinase 1 (TBK1), an upstream key enzyme responsible for IRF-3 activation. Taken together, these findings suggest that TQ can downregulate IRF-3 activation via inhibition of TBK1, which would subsequently decrease the production of type I IFN. TQ also regulated IRF-3, one of the inflammatory transcription factors, providing a novel insight into its anti-inflammatory activities.

## 1. Introduction

Macrophages play a critical role in mediating innate immunity in response to microbial invasion. The cells recognize microbes via pattern recognition receptors (PRRs). Toll-like receptors (TLRs), one of the most characterized PRRs in macrophages, are germline-encoded transmembrane proteins, expressed either at the cell membrane or in endosomes; TLRs can sense various pathogen-associated molecular patterns (PAMPs) [1,2,3,4]. At least 10 types of TLRs have been identified in mammals, and each has a unique ability to recognize different types of PAMPs [3]. Recognition of PAMPs by TLRs results in activation of signaling cascades that ultimately converge to activate transcription factors such as nuclear factor-κB (NF-κB), interferon regulatory factor (IRF), and activator protein-1 (AP-1). Activation of any of these transcription factors results in synthesis and secretion of pro-inflammatory cytokines. Interferon regulatory factor-3 (IRF-3), one of the most studied members of the interferon regulatory transcription factor family, is widely known to play a pivotal role in the activation of interferon genes in responses to viral and bacterial infections [5,6]. Multiple TLRs such as TLR-3, -4, -7, and -9 are known to be involved in the regulation of type I interferons (IFNs) [7]. In particular, lipopolysaccharide (LPS) as a ligand for TLR-4 can be used to trigger IRF-3 activation through the Toll/IL-1R domain-containing adaptor-inducing *IFN-β* (TRIF). Upon stimulation by LPS, TLR-4 recruits the adaptor protein TRIF, which then recruits tumor necrosis factor receptor-associated factor 3 (TRAF3) and the noncanonical IκB kinases, TANK-binding kinase 1 (TBK1) and inhibitor of NF-κB kinase subunit epsilon (IKKε), followed by phosphorylation of IRF-3 at serine/threonine residues of its C-terminal transactivation domain [8]. Phosphorylation of C-terminal serine residues of IRF-3, such as Ser396, induces a conformational change, allowing IRF-3 to dimerize and translocate into the nucleus, where it binds to the promoter of the *IFN-β* gene and stimulates transcription [8,9]. Another finding has suggested that the transforming growth factor beta-activated kinase 1 (TAK1)-c-Jun N-terminal kinases (JNK) cascade is also required for IRF-3 activation [8], providing novel insight into the involvement of mitogen-activated protein (MAP) kinase in innate viral immunity. Inflammation as part of the innate immune response is essential for controlling homeostasis during microbial invasion. In order to maintain balance, the signal transduction of the innate immune response must be under tight regulation. Excessive stimulation could lead to prolonged inflammation, which might lead to a chronic inflammatory disease, such as autoimmune disease, or to cancer. Therefore, developing an agent that can regulate the inflammatory response could provide a way to prevent serious diseases [10].

**Figure 1:**
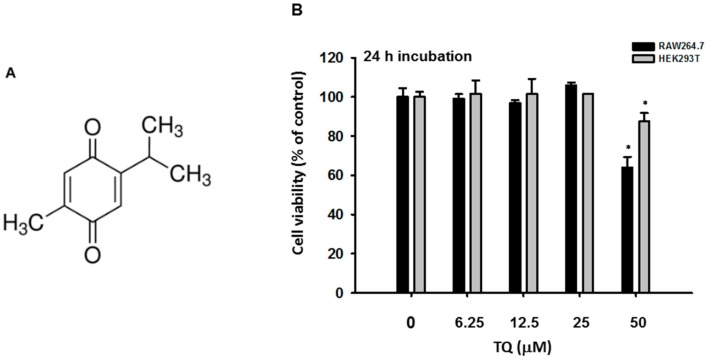
Chemical structure and viability profiles of thymoquinone (TQ). (**A**) Chemical structure of TQ; (**B**) RAW264.7 and HEK293T cells (10^5^ cells/well) were treated with TQ at the indicated doses for 24 h, and cell viability was determined by a 3-(4,5-dimethylthiazol,2-yl)-2,5-diphenyltetrazolium bromide (atetrazole) (MTT) assay. All data are expressed as the mean ± SD. * *p* < 0.05 compared to the normal group.

Thymoquinone (TQ, Figure 1A) is the main constituent of volatile oil derived from black cumin (*Nigella sativa* L.) (Ranunculaceae) seeds, which are known to possess antioxidant, anti-inflammatory, hepatoprotective, and anticancer activities [11]. Studies have suggested that TQ inhibits inflammatory activity by suppressing the interleukin-1 receptor-associated kinase 1 (IRAK1)-linked activator protein (AP)-1 and NF-κB pathways [10]. Although the anti-inflammatory and antiviral activities of this compound have been described, there have been no reports regarding the molecular target of TQ with respect to the IRF-3 pathway. Here, we assessed the ability of TQ to inhibit IRF-3 activation in the LPS-stimulated murine macrophage cell line RAW264.7. Further, we also utilized HEK293T cells for transient expression of enzymes to regulate the IRF-3 signaling pathway as well as IRF-3 luciferase reporter gene assay to gain a better understanding of how TQ could regulate the IRF-3 signaling pathway.

## 2. Results

### 2.1. Effect of Thymoquinone on the mRNA Expression of Interferon Genes in LPS-Stimulated RAW264.7 Cells

**Figure 2:**
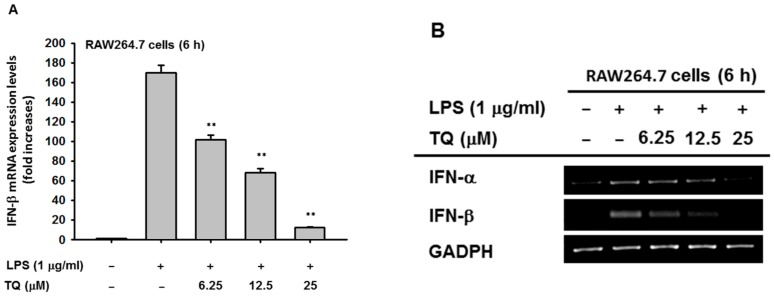
TQ downregulates mRNA expression of type I interferon (IFN)**.** (**A**) RAW264.7 cells (1 × 10^6^ cells/mL) were pretreated with TQ at the indicated doses for 30 min, followed by incubation with lipopolysaccharide (LPS) (1 μg/mL) for 6 h. mRNA expression levels of *IFN-β* and *GAPDH* (as a reference) were determined by real-time PCR; (**B**) mRNA expression levels of *IFN-α*, *IFN-β*, and *GAPDH* (loading control) were determined by semiquantitative reverse transcriptase PCR. All data are expressed as the mean ± SD. ** *p* < 0.01 compared to the control group (LPS alone).

To determine whether TQ is able to alter macrophage-mediated interferon gene expression, we measured the mRNA expression level using semiquantitative reverse transcriptase PCR as well as real-time PCR. We examined the cytotoxicity of TQ (6.25–50 μM) after 24 h in RAW264.7 and HEK293T cells. The viability of both cell types was significantly affected only with TQ 50 μM (Figure 1B). We then investigated the mRNA expression levels of *IFN-β* in RAW264.7 cells pretreated for 30 min with TQ and then stimulated for 6 h with LPS (1 μg/mL). Analysis with real-time PCR showed that LPS induced *IFN-β* mRNA expression. Moreover, TQ decreased mRNA expression of *IFN-β* in a concentration-dependent manner at nontoxic concentrations (Figure 2A). Using semiquantitative RT-PCR, we again found a decrease in interferon mRNA expression. As displayed in Figure 2B, both *IFN-α* and *IFN-β* expression levels decreased in TQ (6.25, 12.5, and 25 μM)-treated cells. TQ weakly decreased *IFN-α* expression level at 12.5 μM but strongly reduced it at 25 μM. Meanwhile, TQ at 25 μM diminished the expression of *IFN-β* mRNA (Figure 2B).

### 2.2. Effect of Thymoquinone on the Transcriptional Activation of IRF-3 

**Figure 3:**
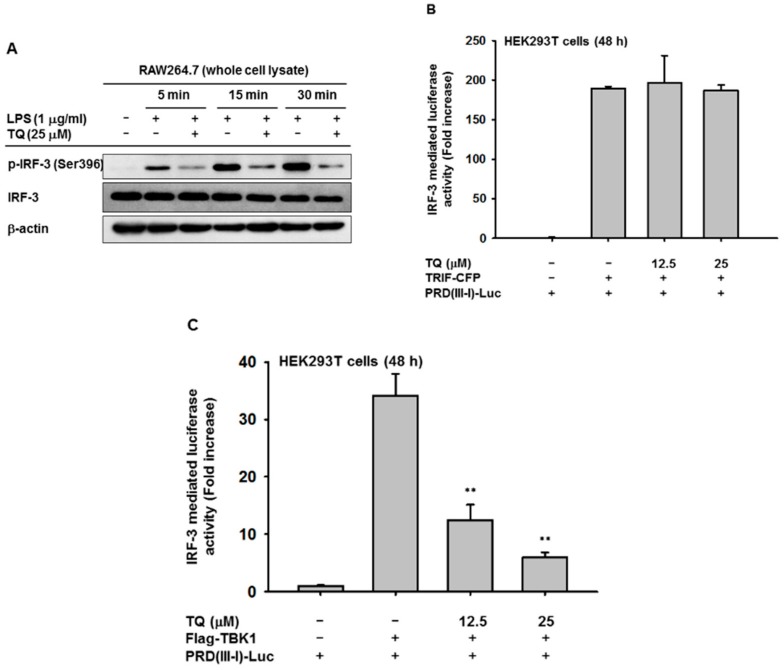
TQ inhibits IRF-3 activation. (**A**) RAW264.7 cells (2 × 10^6^ cells/mL) were pretreated with TQ (25 μM) for 30 min, followed by incubation with LPS (1 μg/mL) for the indicated time points. Phospho- and total levels of IRF-3 in total cell lysate were determined by immunoblotting; (**B**) HEK293T cells (2 × 10^5^ cells/mL) were cotransfected with PRDIII-I-luciferase (0.35 μg/mL), CFP-TRIF (0.35 μg/mL), and β-gal (0.1 μg/mL) for 48 h; (**C**) HEK293T cells (2 × 10^5^ cells/mL) were cotransfected with PRDIII-I-luciferase (0.35 μg/mL), Flag-TBK1 (0.35 μg/mL), and β-gal (0.1 μg/mL) for 48 h. Luciferase activity was measured with a luminometer, and all luciferase reporter gene activities were normalized to β-galactosidase activity. All data are expressed as the mean ± SD of at least three independent experimental replicates. ** *p* < 0.01 compared to the control group.

To confirm the inhibition of interferon genes by TQ related to its regulation of the transcriptional activation of IRF-3, we performed an immunoblotting assay. Immunoblotting was performed with whole cell lysate of RAW264.7 cells that had been pretreated with TQ (25 μM) to analyze whether TQ could alter phosphorylation of IRF-3, a hallmark of initiation of IRF-3 activation. TQ treatment suppressed phosphorylation of IRF-3 at as early as 5 min after LPS stimulation (Figure 3A). IRF-3 activation was also tested with an IRF-3 PRDIII-I-luciferase reporter assay. HEK293T cells were cotransfected with TRIF, an adaptor molecule of TLR-4, or with TBK1, a signaling molecule associated with IRF-3 activation, for 24 h. This was followed by TQ treatment (12.5 and 25 μM) for an additional 24 h without changing the media. As shown in Figures 3B,C, cotransfection either with TRIF or TBK1 triggered increased IRF-3 PRDIII-I-luciferase activity up to 189- or 34-fold, respectively. Treatment with TQ (12.5 and 25 mM) significantly decreased TBK1-mediated IRF-3 PRDIII-I luciferase activity in a concentration-dependent manner, but not TRIF-mediated activity.

### 2.3. Effect of Thymoquinone on Upstream Signaling Events Regulating IRF-3 Activation

**Figure 4:**
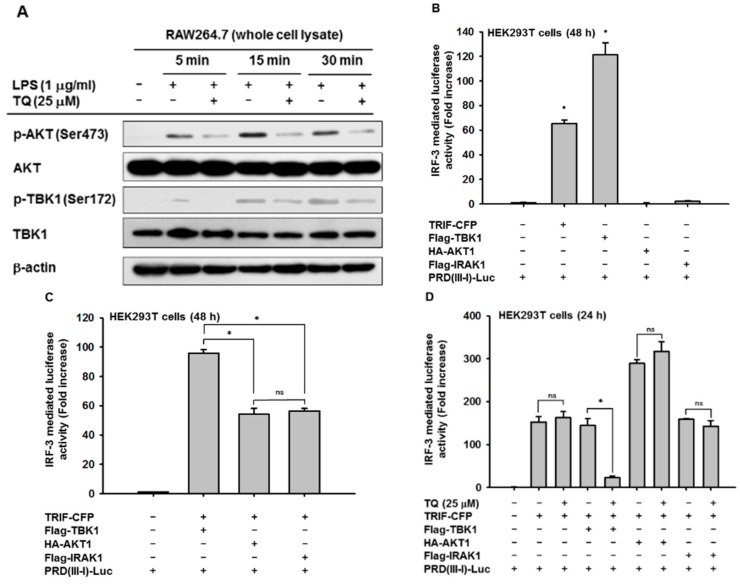
TQ regulates upstream molecules in the IRF-3 pathway. (**A**) RAW264.7 cells (2 × 10^6^ cells/mL) were pretreated with TQ (25 μM) for 30 min, followed by incubation with LPS (1 μg/mL) for the indicated time points. Phospho- and total levels of AKT and TBK1 in total cell lysate were determined by immunoblotting; (**B**,**C**) HEK293T cells (2 × 10^5^ cells/mL) were cotransfected with PRDIII-I-luciferase (0.25 μg/mL), β-gal (0.1 μg/mL), and CFP-TRIF (0.25 μg/mL), HA-AKT1 (0.25 μg/mL), or Flag-IRAK1 (0.25 μg/mL) for 48 h as indicated; (**D**) HEK293T cells (2 × 10^5^ cells/mL) were cotransfected with PRDIII-I-luciferase (0.25 μg/mL), β-gal (0.1 μg/mL), and CFP-TRIF (0.25 μg/mL), HA-AKT1 (0.25 μg/mL), or Flag-IRAK1 (0.25 μg/mL) for 24 h as indicated, followed by TQ (25 μM) treatment for an additional 24 h without changing media. Luciferase activity was measured with a luminometer, and all luciferase reporter gene activities were normalized to β-galactosidase activity. All data are expressed as the mean ± SD of at least three independent experimental replicates. * *p* < 0.05 compared to the control group or normal group. ns: not significant.

Since we observed clear suppression of IRF-3 activation, we further investigated the exact target of TQ inhibition by examining upstream enzymes responsible for IRF-3 activation. The whole cell lysate prepared for the immunoblotting of p-IRF-3 was also used to analyze upstream enzymes associated with IRF-3 activation, including AKT1, TBK1, and IKKε. As displayed in Figure 4A, TQ inhibited the phosphorylation of both AKT and TBK1 by 5 min after LPS stimulation. Meanwhile, inhibition of IKKε was not observed (data not shown), suggesting that AKT and TBK1 are the targets for TQ inhibition in the IRF-3 pathway. Furthermore, we tried to confirm the proposed target by also performing an IRF-3 PRDIII-I-luciferase reporter assay. We assessed whether cotransfection with the proposed target could induce IRF-3-mediated luciferase activity in HEK293T cells, including cotransfection with IRAK1, a known target of TQ with respect to the NF-κB and AP-1 pathways. As shown in Figure 4B, neither AKT1 nor IRAK1 could induce IRF-3-mediated luciferase activity. We confirmed the TQ target once again by cotransfecting either AKT1 or IRAK1 with TRIF. As displayed in Figure 4C, the increase of IRF-3-mediated luciferase activity was not statistically significant between TRIF cotransfected with AKT1 and TRIF cotransfected with IRAK1. The fold induction might be affected by TRIF transfection only, which confirmed that both AKT1 and IRAK1 could not induce IRF-3-mediated luciferase activity. Treatment with TQ (25 μM) was shown to significantly inhibit IRF-3-mediated luciferase activity only when TRIF and TBK1 were cotransfected, but not when TRIF was cotransfected with AKT1 or IRAK1 (Figure 4D).

**Figure 5:**
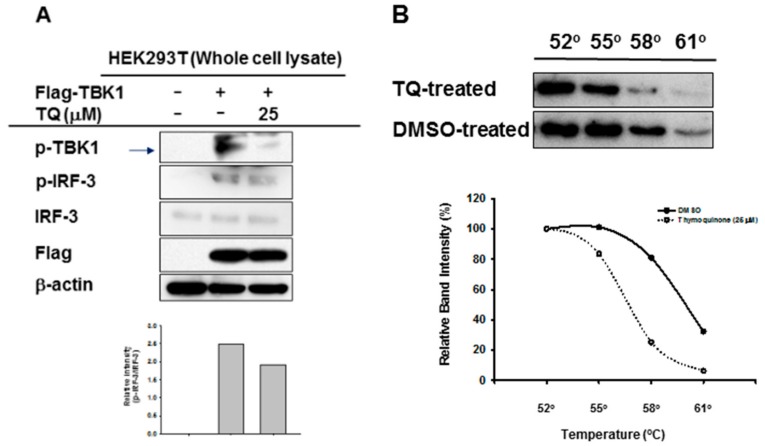
TQ inhibits autophosphorylation of TBK1. (**A**) HEK293T cells (2 × 10^6^ cells/mL) were transfected with Flag-TBK1 (0.8 μg/mL) for 24 h, followed by TQ (25 μM) treatment for an additional 24 h without changing media. The level of p-TBK1, the total level of Flag-TBK1, and the phospho- and total levels of IRF-3 were determined by immunoblotting; (**B**) RAW264.7 cells (2 × 10^6^ cells/mL) were pre-incubated overnight, followed by additional incubation with TQ 25 μM or DMSO for 30 min. Cellular Thermal Shift Assay (CETSA) Classic was performed at four different heating temperatures as indicated. Cell lysates were immunoblotted with a TBK1 antibody.

To evaluate whether TQ could inhibit autophosphorylation of the target enzyme, we performed immunoblotting of whole cell lysate prepared from HEK293T cells overexpressing TBK1. TQ (25 μM) treatment led to reduced phosphorylation of TBK1 in these cells (Figure 5A). Moreover, TQ treatment also reduced phosphorylation of IRF-3 (Figure 5A). Furthermore, to assess the interaction of TQ with TBK1 in intact cells, Cellular Thermal Shift Assay (CETSA) was performed at 4 different temperatures: 52 °C, 55 °C, 58 °C, and 61 °C. As shown in Figure 5B, TQ treatment led to a shift in the thermal stability of the target protein, TBK1. TQ treatment of intact cells tended to lead to destabilization, rather than stabilization, of TBK1.

## 3. Discussion

In this study, we explored the mechanism of the anti-inflammatory activity of TQ with respect to the IRF-3 pathway. We previously reported that TQ could exhibit anti-inflammatory activity by targeting IRAK1, which involves the NF-κB and AP-1 pathways [10]. We believed TQ had promise as an anti-inflammatory agent by targeting multiple pathways in inflammation. Indeed, the significance of the IRF-3 pathway has been reported for inflammation-related diseases such as rheumatoid arthritis, systemic lupus erythematosus, systemic sclerosis, inflammatory bowel disease, chronic obstructive pulmonary disease, and type II diabetes [9,12,13]. In order to confirm our hypothesis that TQ could regulate the IRF-3 pathway, we initially assessed the mRNA expression of interferon genes, as IRF-3 is known to have a central role in their transcription and regulation. By using semiquantitative RT-PCR as well as real-time PCR, we found that TQ can downregulate interferon gene expression of *IFN-α* and *IFN-β* in LPS-stimulated RAW264 cells, as shown in Figures 2A,B. Moreover, TQ reduced the mRNA expression of *IFN-β* in a concentration-dependent manner. This response was not due to nonspecific toxicities, as the viability of the cells was not affected by TQ concentrations up to 25 μM (Figure 1B). This finding implied that TQ could decrease interferon gene expression by regulating the IRF-3-dependent transcription of interferon genes. TQ treatment also reduced *IFN-α* expression but not as strongly as *IFN-β*, which supports our hypothesis, since it is known that IRF-3 is more potent in activating the *IFN-β* rather than the *IFN-α* [14].

Though the pathway leading to IRF-3 activation remains to be elucidated, some of the regulatory molecules have been identified and established. Several of the PRRs identified thus far can mediate the activation of IRF-3, including TLRs, retinoic acid-inducible gene I (RIG-I)-like receptors, and cytosolic DNA sensors such as cyclic GMP-AMP synthase (cGAS) and stimulator of IFN genes (STING) [12]. In macrophages and dendritic cells, TLRs, especially TLR-3 and TLR-4, promote innate viral and bacterial immunity in a TRIF-dependent manner by mediating the production of type I IFN through IRF-3 activation. IFN-β production is triggered by the engagement of TLR-3 by viral double-stranded (ds) RNA/polyinosinic-cytidylic acid (PolyI:C), a dsRNA mimetic, and by the engagement of TLR4 by Gram-negative bacterial cell wall components such as LPS. The mechanism involves recruitment of the adaptor protein TRIF, activation of TBK1 and IKKε, and phosphorylation of IRF-3 [9]. Signaling cascades of the IRF-3 pathway converged to phosphorylate IRF-3, mainly at the serine residues (Ser) 386 and 396. Upon phosphorylation, IRF-3 dimerizes, allowing its translocation into the nucleus where it binds to the promoter and enhancer of IFN-I genes, thereby inducing production of IFN-α and IFN-β.

Therefore, we evaluated IRF-3 activation by immunoblotting of LPS-stimulated RAW264.7 cells to observe the levels of IRF-3 phosphorylated at Ser396. We found that these levels decreased after TQ treatment (Figure 3A), which implies that TQ alters IRF-3 activation through suppression of the phosphorylation of Ser396 on the C-terminal domain of IRF-3. As previously reported, multiple phosphorylation sites have been identified on IRF-3. Activation of IRF-3 appears to be a sequential process in which Ser396 is phosphorylated first, followed by Ser404 and Ser405, thereby priming IRF-3 for phosphorylation on Ser386, which is required for dimerization [15,16]. Interestingly, we found that TQ could suppress the phosphorylation level of IRF-3 Ser396 as early as 5 min after LPS stimulation.

The transcription enhancer of the *IFN-β* gene contains four positive regulatory domains (PRDs), namely, PRD I, II, III, and IV [14], and the promoter region of the *IFN-α* genes contains PRD I- and PRD III-like elements [14]. NF-κB binds to the PRDII element, IRF-3 binds to the adjacent PRDIII and PRDI (referred to as PRDIII-I), and the heterodimeric transcription factor ATF2/c-Jun binds to PRDIV [13]. In our experiment, we transfected cells with the PRDIII-I-luciferase construct to evaluate IRF-3 activation through luciferase activity. We cotransfected the cells with TRIF or TBK1, known to induce IRF-3-mediated luciferase activity. TQ exhibited significant concentration-dependent inhibition of IRF-3-mediated luciferase activity in cells cotransfected with TBK1 but not TRIF (Figures 3B,C). This finding provided evidence that TQ could alter the promoter activity of IRF-3, thereby regulating IRF-3 activation. While the specific inhibition occurred only with TBK1 cotransfection, it is still unclear why the inhibition did not occur when using TRIF as an inducer. It is possible that TQ inhibition is specific for TBK1 binding to the IRF-3 promoter and independent of direct binding of TRIF to the IRF-3 promoter. TRIF contains an N-terminal fragment containing ~540 amino acids, which has been shown to bind IRF3 and activate the interferon promoter when transiently expressed in HEK293T cells [17]. Further study is required to address this issue.

Besides TRIF, other upstream molecules have been identified as being responsible for IRF-3 activation, including TRAF3, TANK, AKT, TBK1, IKKε, and IKKβ [18,19,20]. By using an immunoblotting assay, we checked the protein expression of several of these molecules. In agreement with another report, stimulation with LPS in RAW264.7 increased the catalytic activity of TBK1, as was shown by phosphorylation at Ser172 in RAW264.7 cells, which reached a maximum before 30 min after LPS stimulation [21]. The protein levels of p-AKT and p-TBK1 were suppressed by TQ treatment after 5 min of LPS stimulation in RAW264.7 cells (Figure 4A). Meanwhile, no inhibition was observed in the phosphorylation of IKKε (data not shown). These data suggest that AKT and TBK1 are targeted by TQ in the IRF-3 pathway. Indeed, AKT has been reported to be a downstream component of TBK1 and TRIF, which play roles in IRF3 activation [19]. Therefore, decreasing the activation of AKT is thought to result from the suppression of TBK1 phosphorylation. We investigated whether this inhibition is associated with IRAK1, since IRAK1 is known to be targeted by TQ in the NF-κB and AP-1 pathways. We cotransfected the cells with construct genes for each proposed target, including TBK1, AKT1 (one of the major AKT isoforms), and IRAK1, with PRDIII-I-luciferase in an IRF-3-mediated luciferase assay. The results demonstrated that neither AKT1 nor IRAK1 alone could increase IRF-3 PRDIII-I-mediated luciferase activity (Figure 4B). The same results were observed when we cotransfected AKT1 or IRAK1 with a TRIF construct gene (Figure 4C). Meanwhile, there was no significant difference between the TQ treatment group and the nontreated group when PRDIII-I-luciferase was cotransfected with TRIF and IRAK1 (Figure 4D), suggesting that the inhibition of TBK1-induced promoter activity of IRF-3 by TQ is uncoupled with its effect on IRAK1 inhibition.

It has been reported that phosphorylation of TBK1 at Ser172 is at least partly an intermolecular autophosphorylation event [21,22,23]. In agreement with previous reports, when we overexpressed TBK1 in HEK293T cells, we found expression of phosphorylated TBK1. Moreover, treatment with TQ decreased protein expression of phosphorylated TBK1 (Figure 5A). As expected, TQ’s inhibition of TBK1 activation resulted in the inhibition of IRF-3 activation as well (Figure 5A). This indicates that TQ acts to downregulate IRF-3 partly by inhibition of TBK1 autophosphorylation. To understand the interaction of TQ with its target protein TBK1, we performed a CETSA. Upon heat treatment at different temperatures, the target protein unfolded and aggregated. Soluble thermostabilization of TBK1 by TQ was then analyzed by western blotting. As shown in Figure 5B, TQ shifted the thermal stability of TBK1. Furthermore, that shift led to its destabilization rather than stabilization. To our knowledge, this is not the first time the CETSA has shown such destabilization. In other reports, drugs that act as receptor antagonists can reverse agonist-mediated receptor thermal stabilization, as shown in a CETSA [24]. TBK1 is a serine/threonine protein kinase, which is activated by autophosphorylation or by phosphorylation by other kinases. TBK1 contains a kinase domain (KD) that houses its catalytic activity, a ubiquitin-like domain (ULD), a dimerization domain (DD), and a small protein interaction module at the C-terminus termed as an adaptor-binding motif [25,26]. Moreover, known TBK1 inhibitors (BX795, MRT67307, and MRT67215) have been reported to compete with ATP, since they bind to KD in a similar manner by positioning in the cleft between the N- and C-lobes and occupying several subregions of the ATP binding site, thus stabilizing the catalytic spine of the kinase [26]. Structural studies have reported that, although the TBK1 ULD and DD do not physically occlude the kinase active site or allosterically control the catalytic competency of the KD, these accessory domains autoinhibit TBK1 by restricting self-associations that lead to autophosphorylation of the TBK1 activation loop [25]. Further studies are required to increase our understanding of the interaction of TBK1 with TQ: for example, whether TQ acts as an ATP competitor on TBK1. In addition, based on our findings, the role of TBK1 inhibition by TQ with respect to the endoplasmic reticulum (ER) signaling pathway could be addressed in the future studies. Since it is known that TBK1 is highly associated with ER-resident adaptor protein STING, there seems to be a possibility that TQ might functionally regulate the activity of STING to promote the phosphorylation of IRF-3 [27]. Therefore, it would be very interesting to explore the correlation of TBK1 inhibition by TQ and ER stress-mediated apoptosis which might be contributing to the anticancer activity of TQ.

**Figure 6:**
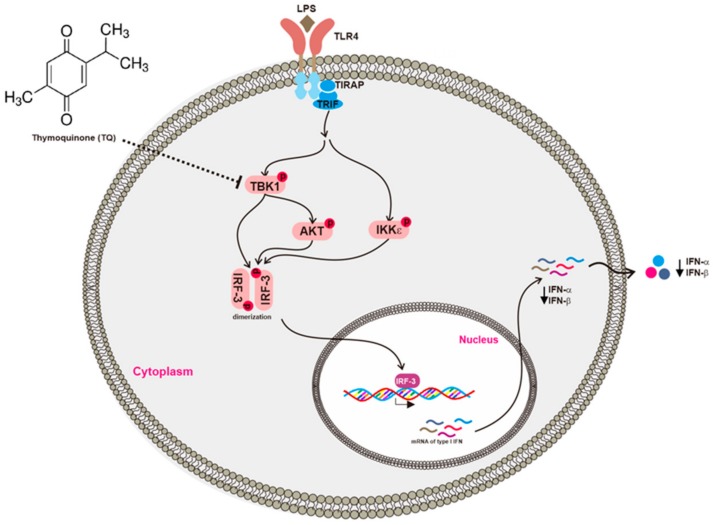
Putative inhibitory activity of TQ with respect to the IRF-3 pathway. TQ downregulated type I IFN by targeting TBK1, which suppressed IRF-3 activation.

Conclusively, the present study aimed to clarify the anti-inflammatory activity of TQ with respect to the IRF-3 pathway. TQ inhibited the activation of TBK1, one of the critical upstream enzymes responsible for IRF-3 activation, and thereby suppressed the expression of type I IFNs including *IFN-α* and *IFN-β* as summarized in Figure 6. These findings suggest that TQ can also regulate IRF-3, one of the inflammatory transcription factors, providing a novel insight into its anti-inflammatory activities.

## 4. Materials and Methods 

### 4.1. Materials

TQ (purity: 99%), polyethylenimine (PEI), 3-(4,5-dimethylthiazol,2-yl)-2,5-diphenyltetrazolium bromide (atetrazole) (MTT), sodium dodecyl sulfate (SDS), dimethyl sulfoxide (DMSO), and lipopolysaccharide (LPS, *E. coli* 0111:B4) were purchased from Sigma-Aldrich Co. (St. Louis, MO, USA). Roswell Park Memorial Institute (RPMI) 1640, Dulbecco’s modified Eagle’s medium (DMEM), penicillin–streptomycin, and trypsin were purchased from HyClone (Logan, UT, USA). Phosphate-buffered saline (PBS) was purchased from Capricorn Scientific GmbH (Ebsdorfergrund, Germany). Fetal bovine serum (FBS) was purchased from Biotechnics Research, Inc. (Irvine, CA, USA). TRIzol reagent was purchased from MRCgene (Cincinnati, OH, USA). RAW264.7 and HEK293T cells were purchased from American Type Culture Collection (ATCC) (Rockville, MD, USA). Primers used for semiquantitative reverse transcriptase (RT)-polymerase chain reaction for *IFN-β* were purchased from Bioneer Corp. (Daejeon, Korea). The rest of the primers used in this study were purchased from Macrogen Inc. (Seoul, Korea). PCRBIO HS Taq PreMix and qPCRBIO SyGreen Blue Mix Lo-ROX were purchased from PCR Biosystems Ltd. (London, UK). Phosphospecific or total protein antibodies raised against IRF-3, protein kinase B (AKT), TBK1, Flag (DYKDDDDK) tag, and β-actin were obtained from Cell Signaling Technology (Beverly, MA, USA). Constructs for signaling proteins including cyan fluorescent protein (CFP)–TIR-domain-containing adapter-inducing interferon-β (TRIF), Flag-IRAK1, Flag-TBK1, hemagglutinin (HA)-AKT1, and interferon-β PRDIII-I-luciferase reporter plasmid were used as reported previously [28,29,30].

### 4.2. Cell Culture and Drug Preparation

A murine macrophage cell line (RAW264.7) and a human embryonic kidney cell line (HEK293T) were cultured in RPMI 1640 and DMEM media, respectively, which were each supplemented with 100 U/mL of penicillin, 100 μg/mL of streptomycin, and 2 mM l-glutamine. In addition, the RPMI 1640 and DMEM media were supplemented with 10% and 5% heat-inactivated FBS, respectively. Both of the cell lines were grown at 37 °C under 5% CO_2_ in a humidified incubator. The stock solutions of TQ were prepared by diluting TQ powder with 100% DMSO in an amber microcentrifuge tube.

### 4.3. Cell Viability Assay

The cytotoxic effect on RAW264.7 and HEK293T cells was evaluated by treating cells (10^5^ cells/well) with TQ (0, 6.25, 12.5, 25, and 50 μM) for 24 h, after they were pre-incubated for 18 h in 96-well plates as reported previously [31]. The treatment was followed by a conventional MTT assay. Specifically, 100 μL of cell culture was incubated with 10 μL of MTT solution (10 mg/mL in PBS pH 7.4) for 3 h at 37 °C. The reaction was stopped by adding 100 μL of MTT stopping solution (15% sodium dodecyl sulfate), followed by incubation for 8 h at 37 °C. The absorbance was then measured at 570 nm using a Synergy HT Multi-Mode Microplate Reader (BioTek Instruments GmbH, Bad Friedrichshall, Germany).

### 4.4. mRNA Analysis by Semiquantitative Reverse Transcriptase (RT)-Polymerase Chain Reaction (PCR) and Real-Time PCR (qPCR)

In order to analyze the mRNA expression levels of *IFN-α* and *IFN-β*, total RNA was prepared from RAW264.7 cells (1 × 10^6^ cells/mL) that had been pretreated with TQ (0, 6.25, 12.5, and 25 μM) for 30 min and then incubated with LPS (1 μg/mL) for 6 h. Total RNA was isolated using TRIzol reagent according to a previous method [32], and then 1 μg of total RNA was immediately used for cDNA synthesis using a cDNA synthesis kit (Thermo Fisher Scientific, Waltham, MA, USA) according to the manufacturer’s instructions. Semiquantitative RT-PCR was performed using the following mixture: 2×PCRBIO HS Taq PreMix 10 μL, forward and reverse primer (10 μM) 2 μL each, cDNA 2 μL, and diethyl pyrocarbonate-treated water at 6 μL per 20 μL of reaction volume. The PCR reaction was conducted under the following conditions: 15 s denaturation time at 95 °C, 15 s annealing time at 55 °C (*IFN-α* and *GAPDH*) or 62 °C (*IFN-β*), and 60 s extension time at 72 °C, for 30 cycles. After completion of PCR, a 10 μL aliquot was subjected to agarose gel electrophoresis. mRNA expression was quantified by real-time PCR using qPCRBIO SyGreen Blue Mix Lo-ROX according to the manufacturer’s instructions (PCR Biosystems Ltd., London, UK), using a real-time C1000 thermal cycler (Bio-Rad Laboratories Inc., Hercules, CA, USA) with some modifications. The thermal cycler conditions for real-time PCR were as follows: 10 s denaturation time at 95 °C, 10 s annealing time at 58 °C, and 60 s extension time at 72 °C, for 39 cycles, followed by detection of fluorescent product at the last step of each cycle. The primer sequences used in this study are listed in Table 1.

### 4.5. Luciferase Reporter Gene Assay

HEK293T cells (2.5 × 10^5^ cells/mL) were cotransfected with interferon-β PRDIII-I-luciferase reporter plasmid and β-galactosidase and other constructs for signaling proteins such as CFP-TRIF, Flag-TBK1, HA-AKT1, Flag-IRAK1, or empty vector for 24 h, using PEI, in 24-well plates as reported previously [33]. The cells were then treated with or without TQ (12.5 and 25 μM) for 24 h and then lysed by freezing and thawing. An aliquot of cell lysate was reacted with luciferase substrate, followed by a luminescence reading. Another aliquot of cell lysate was reacted with β-galactosidase substrate, followed by an absorbance reading at 405 nm. Luminescence and absorbance were measured using a Synergy HT Multi-Mode Microplate Reader (BioTek Instruments GmbH, Bad Friedrichshall, Germany). All luciferase activities were normalized to β-galactosidase activity.

### 4.6. Preparation of Whole Cell Lysates and Immunoblotting 

RAW264.7 cells were pretreated with TQ 25 μM, followed by LPS (1 μg/mL) stimulation for 5, 15, and 30 min. HEK293T cells were independently transfected with Flag-TBK1 or empty vector for 24 h, followed by TQ 25 μM treatment for an additional 24 h. Whole cell lysates were prepared as described previously [29]. Immunoblotting of phosphorylated or total levels of IRF-3, TBK1, and AKT was conducted as described previously [29]. β-actin was used as a loading control.

### 4.7. Cellular Thermal Shift Assay (CETSA) 

The interaction of TQ with the proposed target protein (TBK1) in intact cells was examined using CETSA classic as reported previously [34]. RAW264.7 cells (2 × 10^6^ cells/mL) were pre-incubated overnight and then incubated with TQ 25 μM or DMSO vehicle for 30 min. The cells were collected in PBS and pelleted by centrifugation. All of the supernatant was discarded, and each pellet was resuspended with PBS supplemented with protease inhibitor. Each cell suspension (DMSO-treated and TQ-treated) was further distributed into 4 different PCR tubes, with 100 μL of cell suspension in each tube. Each tube was marked with the designated temperature (52, 55, 58, 61 °C). Before heat treatment, the tubes were kept at room temperature. Heat treatment was performed using a SimpliAmp Thermal Cycler (Applied Biosystems, Foster City, CA, USA) with VeriFlex tools, providing a configuration of independent temperature zones. All of the PCR tubes were heated at the designated temperature for 3 min, incubated at room temperature for 3 min, and immediately snap-frozen in liquid nitrogen. The cells were further lysed over 3 freeze–thaw cycles by alternating liquid nitrogen and a heating block set at 25 °C. Cell lysates were subjected to immunoblotting as described previously [29].

### 4.8. Statistical Analysis

All data in this study are presented as the mean and standard deviation of at least three independent replicate experiments. Statistical comparisons were analyzed using either analysis of variance (ANOVA)/Scheffe’s post hoc test or the nonparametric Kruskal–Wallis/Mann–Whitney test. A value of *p* < 0.05 was considered to be a statistically significant difference. All statistical analysis was performed using SPSS software (IBM Corporation, Armonk, NY, USA).

## Figures and Tables

**Table 1:** Primer sequences used for PCR in this study.

PCR Type	Name	Sequence (5′–3′)	
Reverse Transcriptase PCR	*IFN-α*	Forward Reverse	CAACACCTACACAGGTTACC AGTGGCTTCCCAGATGTTCC
	*IFN-β*	Forward Reverse	TCCAAGAAAGGACGAACATT TGAGGACATCTCCCACGTCA
	*GAPDH*	Forward Reverse	ACCACAGTCCATGCCATCAC CCACCACCCTGTTGCTGTAG
Real Time PCR	*IFN-β*	Forward Reverse	AAGAGTTACACTGCCTTTGCTATC CACTGTCTGCTGGTGGAGTTCATC
	*GAPDH*	Forward Reverse	CAATGAATACGGCTACAGCA AGGGAGATGCTCAGTGTTGG
